# Stacked generalization as a computational method for the genomic selection

**DOI:** 10.3389/fgene.2024.1401470

**Published:** 2024-07-10

**Authors:** Sunhee Kim, Sang-Ho Chu, Yong-Jin Park, Chang-Yong Lee

**Affiliations:** ^1^ The Department of Industrial Engineering, Kongju National University, Cheonan, Republic of Korea; ^2^ The Department of Plant Resources, Kongju National University, Yesan, Republic of Korea

**Keywords:** stacked generalization, ensemble method, base models, meta-model, genomic selection, non-inferiority testing, overfitting

## Abstract

As genomic selection emerges as a promising breeding method for both plants and animals, numerous methods have been introduced and applied to various real and simulated data sets. Research suggests that no single method is universally better than others; rather, performance is highly dependent on the characteristics of the data and the nature of the prediction task. This implies that each method has its strengths and weaknesses. In this study, we exploit this notion and propose a different approach. Rather than comparing multiple methods to determine the best one for a particular study, we advocate combining multiple methods to achieve better performance than each method in isolation. In pursuit of this goal, we introduce and develop a computational method of the stacked generalization within ensemble methods. In this method, the meta-model merges predictions from multiple base models to achieve improved performance. We applied this method to plant and animal data and compared its performance with currently available methods using standard performance metrics. We found that the proposed method yielded a lower or comparable mean squared error in predicting phenotypes compared to the current methods. In addition, the proposed method showed greater resistance to overfitting compared to the current methods. Further analysis included statistical hypothesis testing, which showed that the proposed method outperformed or matched the current methods. In summary, the proposed stacked generalization integrates currently available methods to achieve stable and better performance. In this context, our study provides general recommendations for effective practices in genomic selection.

## 1 Introduction

Genomic selection (GS), first introduced in Ref. (Meuwissen et al., 2001), is a methodology for improving selection and breeding processes for plants and animals. It involves the identification of patterns or associations between genetic markers and observed trait values. GS uses genome-wide markers, typically single nucleotide polymorphisms (SNPs), extracted from samples to compute genomic estimated breeding values for a particular trait of interest. In GS, the data set containing observable genotypic and phenotypic information is used to train a model (i.e., estimate the model parameters) and predict the breeding value or related quantities based on the trained model and the genotypic information obtained from the markers. By using GS, breeders can assess the likelihood that samples will transmit desirable traits to their progeny. As a result, GS is proving to be a valuable tool for the genetic improvement of plants and animals by increasing the selection accuracy for specific traits, such as yield and disease resistance.

Various statistical and machine learning methods are available for GS (de Los Campos et al., 2013; Crossa et al., 2017). These methods can be broadly categorized into linear and nonlinear models (Howard et al., 2014). Nonlinear models, such as support vector machines, artificial neural networks, and random forests, fall under the machine learning methods within GS (Jubair and Domaratzki, 2023). On the other hand, linear models can be further subdivided into linear mixed models and Bayesian models. Bayesian models such as BayesA and BayesB (Meuwissen et al., 2001) are representative examples, while linear mixed models include rrBLUP (Endelman, 2011) and gBLUP (Clark and van der Werf, 2013). The linear models differ primarily in their assumptions about the distribution and variance of the marker effects.

Numerous efforts have been made to evaluate the performance of different models, especially within the linear model domain, under different scenarios. Comparative analyses between gBLUP and Bayesian models have been conducted using both real and simulated data sets (Haile et al., 2020; Hong et al., 2020; Nsibi et al., 2020; Zhu et al., 2021). In addition, evaluations of other BLUP variants, such as cBLUP and sBLUP, have been conducted alongside Bayesian models on various types of data (Meher et al., 2022). A study comparing the performance of rrBLUP with BayesB and Bayesian Lasso (or BayesL) has been conducted on various traits of wheat, barley, and maize (Heslot et al., 2012). Beyond model comparisons, the cross-validation method has been proposed to measure differences in model accuracy (Schrauf et al., 2021). However, it has been observed that no single model is universally superior under different circumstances (Pérez and de Los Campos, 2014). Instead, the effectiveness of models depends on the specific characteristics of the data and the nature of the prediction task at hand (Azodi et al., 2019). This underscores the challenge of conclusively determining which model consistently outperforms others, and points to the need for extensive validation in different contexts.

Selecting an appropriate method for GS is challenging due to the wide variety of models available. The selection process is complex and influenced by many factors, including the characteristics of the population being studied and the complexity of the traits of interest. Determining an appropriate method often involves a trial-and-error approach, as the suitability of a method may vary depending on the context and data availability. Therefore, making an informed choice requires a thoughtful and iterative strategy, coupled with a deep understanding of the problem. This process becomes particularly critical when there is a lack of theoretical and/or experimental confidence in the chosen method.

In this study, we propose a computational method that is conceptually different from conventional methods. Instead of selecting an appropriate method through a comparative performance analysis, we advocate an integration strategy. This involves combining multiple models to exploit the strengths of each, leading to improved and more robust results. This approach follows the principles of ensemble methods in machine learning (Rokach, 2010; Mienye and Sun, 2022). By leveraging the collective knowledge of multiple models, ensemble methods become powerful tools for improving performance, especially when individual models have distinct strengths and weaknesses. As a result, ensemble methods have the potential to deliver superior performance compared to individual models, albeit at the cost of increased computational time.

As our chosen ensemble method, we implemented the stacked generalization (Wolpert, 1992), commonly known as stacking. Stacking involves the integration of multiple models, called base models, along with an additional model, called the meta-model. The base models generate predictions for the data, and the meta-model is tasked with learning how to optimally combine these predictions to produce the final predictions. We selected six base models derived from the linear mixed and Bayesian models widely used in GS. To effectively combine the results of the base models, we used a neural network of a multi-layer perceptron as our meta-model. With this, we investigated the possibility of stacking as a method for GS.

The proposed stacking was applied to open-access resources of rice, maize, barley, mice, and millet. Our analysis involved comparing the performance of the stacking model with that of its constituent base models. To compare the performance of the models, we evaluated quantities such as overfitting and mean squared error (MSE) between observed and predicted phenotype values, which served as a measure of the robustness and prediction accuracy of the models. We also performed the hypothesis tests for prediction accuracy between the proposed model and each base model. Our results showed that the proposed model generally outperformed the base models in different scenarios. As another advantage of the proposed model, we highlighted its effectiveness in reducing overfitting. From these results, we conclude that the proposed model emerges as a promising tool for efficient practices in GS.

## 2 Materials and methods

### 2.1 Data acquisition and preparation

We used the open resource of genomic data sets from different species: rice, barley, maize, mice, and millet. The rice data set, the 44K_SNP (Zhao et al., 2011), consists of SNP genotype and phenotype information of rice accessions. It consists of 36,901 SNPs in 413 different rice accessions, each phenotyped for 34 traits, and can be downloaded from Ref. (Zhao, 2024). under the title “44K SNP set”. We used 30 quantitative (or numerical) traits. We pre-processed the data set by eliminating SNPs and accessions with missing genotype and/or phenotype values. The pre-processed data consists of 3,686 SNPs from 198 accessions with 30 quantitative traits for each accession. A list of the 30 quantitative traits with their abbreviations is provided in Supplementary Table S1.

The barley data set consists of 7,864 SNPs in 310 samples, each phenotyped for eight traits (Nielsen et al., 2016). The data are available at Ref. (Nielsen, 2024). We also pre-processed the data set by eliminating SNPs and accessions with missing genotype and/or phenotype values. The pre-processed data consists of 5,160 SNPs from 307 accessions. A list of eight quantitative traits with their abbreviations is provided in Supplementary Table S2.

The maize data set consists of 83,153,144 SNPs in 282 samples, each phenotyped for 11 traits (Peiffer et al., 2014). The data can be downloaded from https://www.panzea.org/phenotypes under the title “Maize 282 association panel phenotypes”. We pre-processed the data set by eliminating SNPs and accessions with missing genotype and/or phenotype values. The pre-processed data consists of 45,438 SNPs from 262 accessions. A list of the 11 quantitative traits with their abbreviations is provided in Supplementary Table S3.

The mouse data set consists of 10,346 SNPs in 1,814 samples, each phenotyped for 25 traits (Pérez and de Los Campos, 2014). It can be downloaded at Ref. (Pérez, 2024). We pre-processed the data set by eliminating samples with missing phenotype values, resulting in 1,181 samples. We also eliminated five traits that had too many missing phenotype values and used 20 traits. A list of the 20 quantitative traits with their abbreviations is provided in Supplementary Table S4.

The millet data set consists of 161,562 SNPs in 827 samples, each phenotyped for 12 traits (Wang et al., 2022). It can be downloaded at Ref. (CropGS-Hub, 2024). We pre-processed the data set by eliminating samples with missing phenotype values, resulting in 13,807 SNPs from 827 samples. A list of the 12 quantitative traits with their abbreviations is provided in Supplementary Table S5.

### 2.2 Base models and meta-model

We selected the base models from linear parametric models representing phenotypes with genetic markers. For a given set of n samples and p markers, the linear model for the i th phenotype $y_{i}$ is expressed as:

$$y_{i}=\mu +\overset{p}{\underset{j=1}{\sum }}Z_{ij}\beta_{j}+e_{i},\;\;\text{where}\;\;i=1,2,\ldots ,n\;\;\text{and}\;\;j=1,2,\ldots ,p.$$ (1)

Here, μ is the overall mean, $Z_{ij}$ is a n×p genotype matrix (e.g., −1, 0, 1 for aa , Aa , AA genotypes, respectively), and $\beta_{j}$ is the j th marker effect. In addition, the residual $e_{i}$ is assumed to follow $e_{i}∼N\left(0,\sigma_{e}^{2}\right)$ .

In GS, a common challenge arises when the number of markers p (e.g., the number of SNPs) exceeds the sample size n , known as the “small n , big p ” problem. In this n<p scenario, the maximum likelihood estimator for $\beta =\left\{\beta_{1},\beta_{2},\ldots ,\beta_{p}\right\}$ in Eq. 1 is neither unique nor unbiased. Consequently, the maximum likelihood approach is inappropriate for estimating β . To overcome this, an alternative method introduces an additional assumption regarding marker effects. Two common approaches are best linear unbiased prediction (BLUP) and Bayesian models. Both methods assume probability distributions for marker effects but differ in the interpretation of probability and the approach to parameter inference (Gianola, 2013).

Bayesian models introduce regularization by incorporating appropriate priors that impose constraints on marker sizes. These models assume a prior distribution for marker effects, and different choices of priors lead to different Bayesian models. Once a prior is chosen, the posterior estimate of the marker effects $\beta_{j}$ in Eq. 1 can be computed using the likelihood derived from the data set. For a comprehensive overview and brief historical context of Bayesian models, see Refs. (Robinson, 1991; Gianola et al., 2009).

The BLUP model, on the other hand, assumes that the marker effects are drawn from a distribution with a known variance component, resulting in the linear mixed model (Henderson, 1977). It can be written as

$$y_{i}=\overset{p}{\underset{j=i}{\sum }}X_{ij}\beta_{j}+\overset{p}{\underset{j=1}{\sum }}Z_{ij}u_{j}+e_{i}.$$ (2)

Here, $y_{i}$ is the phenotype of the i th sample, $\beta_{j}$ are fixed effects, $u_{j}$ are random effects, and $Z_{ij}$ is the design matrix. The $\sum_{j=1}^{p}X_{ij}\beta_{j}$ replaces the overall mean μ in Eq. 1 to include all fixed effects. The linear mixed model assumes that the random effects are $u_{j}∼N\left(0,K\right)$ and the residuals are $e_{i}∼N\left(0,R\right)$ . Note that the $\beta_{j}$ for the fixed effects in Eq. 2 should not be confused with the $\beta_{j}$ representing the marker effects in Eq. 1. This potential source of confusion stems from conventional notations used in the statistical and genomic selection literature.

To build the stacking model, we selected six base models based on their prevalence and the diversity they contribute to GS. Our selections included rrBLUP and gBLUP from the linear mixed models; BayesA, BayesB, BayesC, and BayesL from the Bayesian models. These models were chosen to capture the range of approaches available for GS, with an emphasis on their application to real-world data sets (Cui et al., 2020; Diaz et al., 2021; Budhlakoti et al., 2022). gBLUP represents a model that does not rely on estimating marker effects, while rrBLUP estimates marker effects using both linear and penalized parameters. The two models are considered equivalent under certain conditions (Goddard, 2009). From a Bayesian perspective, Bayesian models fall into four categories: Gaussian, spike-slab, thick-tail, and mass-point slab (de Los Campos et al., 2013). We exclude the Gaussian prior because its posterior mean is equivalent to rrBLUP. We also omit the spike-slab models because they can be viewed as a combination of the thick-tail and mass-point slab models. Among the Bayesian models, BayesA and BayesL fall under the thick-tail model, while BayesB and BayesC fall under the mass-point slab model. Table 1 lists the base models and their estimates or priors used in this study.

**TABLE 1:** A list of selected base models and their estimate (or prior) of the marker effects, together with the corresponding hyper-parameters. In the hyper-parameters column, ν , π , and $S_{\beta }$ are the degree of freedom, the proportion of non-null effects, and the scaled parameter, respectively. The k in gBLUP is a quantity related to the allele frequency.

Model	Estimate (or prior) of marker effects	Hyper-parameters
rrBLUP	$\hat{u}={\left(Z^{T}Z+\sigma_{e}^{2}\sigma_{u}^{-2}I\right)}^{-1}Z\left(y-X\hat{\beta }\right)$	
gBLUP	$\hat{m}={\left(1+\sigma_{e}^{2}\sigma_{u}^{-2}G^{-1}\right)}^{-1}\left(y-X\hat{\beta }\right)$ , where m=Zu and $G=kZZ^{T}$	
BayesA	$\beta_{j}|\nu ,S_{\beta }∼t\left(\nu ,S_{\beta }\right)$	ν , $S_{\beta }$ , $S_{\beta }∼\Gamma \left(r,s\right)$
BayesB	$\beta_{j}|\nu ,S_{\beta }∼\left\{\begin{array}{cc} t\left(\nu ,S_{\beta }\right),\; & \text{with probability }\pi \\ 0,\; & \text{with probability }1-\pi \end{array}\right.$	π∼Beta(α,β) , ν , $S_{\beta }∼\Gamma \left(r,s\right)$
BayesC	$\beta_{j}|\sigma_{\beta }^{2}∼\left\{\begin{array}{cc} N\left(0,\sigma_{\beta }^{2}\right),\; & \text{with probability }\pi \\ 0,\; & \text{with probability }1-\pi \end{array}\right.$	π∼Beta(α,β) , $\sigma_{\beta }^{2}∼\chi^{-2}\left(\nu ,S_{\beta }\right)$
BayesL	$\beta_{j}|\sigma_{\beta }^{2}∼L\left(\lambda^{2}\right)$	$\lambda^{2}∼\Gamma \left(r,s\right)$

Given a specific prior, we derive the posterior distribution of a marker effect by applying Bayes’ theorem, incorporating the likelihood from the available data. Since the posterior cannot be typically expressed in closed form, numerical evaluation becomes necessary. A widely used method for this purpose is the Gibbs sampling method (López et al., 2022), a Markov chain Monte Carlo algorithm designed to generate a sequence of observations that allow an approximation of the joint distribution. Gibbs sampling iteratively generates samples from the conditional distributions of each parameter. After obtaining a sample from the posterior distribution, parameter estimates are often derived by averaging these sample values. In our implementation, we used the R package “Bayesian Generalized Linear Regression” (BGLR) (Pérez and de Los Campos, 2014) for the Bayesian models and the R package “rrBLUP” (Endelman, 2011) for the linear mixed models.

For the meta-model, we used a neural network of a multilayer perceptron, which consists of one hidden layer of nodes in addition to input and output layers. The multilayer perceptron is a feed-forward neural network in which all nodes in the previous layer are connected to each node in the current layer. The input is the predictions from the base models and the output is the predicted phenotype. The predictions generated by six base models consist of six nodes in the input layer, and the final prediction is obtained from one node in the output layer.

### 2.3 Model selection

Because the number of markers exceeds the number of samples, most GS models have penalized parameters that control the degree of model fit to the data. In the rrBLUP and gBLUP, the penalized parameters of the variance components can be estimated by minimizing the restricted maximum likelihood criterion. In the Bayesian models, however, the penalized parameters that control the nature and extent of the regularization are essentially unknown. Each prior used in this study is specified by one or more penalized parameters, some of which are given as probability distributions of unknown parameters. Thus, in the Bayesian models, the penalized parameters are hyper-parameters that affect their performance in fitting real data. For example, in BayesA and BayesB models, the hyper-parameters are the degrees of freedom and the scale parameter of a scaled t distribution. The hyper-parameters control the type and degree of shrinkage (BayesA and BayesL) or variable selection (BayesB and BayesC). The hyper-parameters of the Bayesian model are listed in Table 1.

We followed the rules built into the R package BGLR for the penalized hyper-parameters setting. The default rule splits the variance of the phenotype into components attributable to the model residuals and the marker effects. The package allows for control of the proportion $R^{2}$ of the phenotypic variance that is expected to be explained by the marker effects. To find an optimal $R^{2}$ , we ran a 5-fold CV with a grid of values for $R^{2}$ to examine the change in the performance. For hyper-parameters other than the penalized parameters, we used the default values given in BGLR. For example, the proportion of non-null effects is set to π=0.5 in a-prior; the shape parameter of the gamma density for BayesL is set to $\lambda^{2}=1.1$ .

Our model selection process for the meta-model is significantly simpler than current deep learning architectures because we use a simple perceptron with one hidden layer in addition to input and output layers. Through experimentation, we found that using the sigmoid activation function for the hidden layer yielded slightly better results compared to alternatives such as ReLU. In addition, we explored several optimizers, including stochastic gradient descent, Adagrad, RMSprop, and Adam (Choi et al., 2020). While the variance in performance among the optimizers was negligible, we observed that Adam performed optimally with a learning rate set to 0.001. In addition, our experiments showed that the choice of mini-batch size, approximately 50, and epochs, more than 400, had minimal impact on the results. Furthermore, we found that the number of nodes in the hidden layer was relatively insensitive, provided it exceeded the size of the input nodes. Given our task of predicting a continuous phenotype, we used the mean squared error as our preferred loss function.

### 2.4 Stacked generalization

**FIGURE 1:**
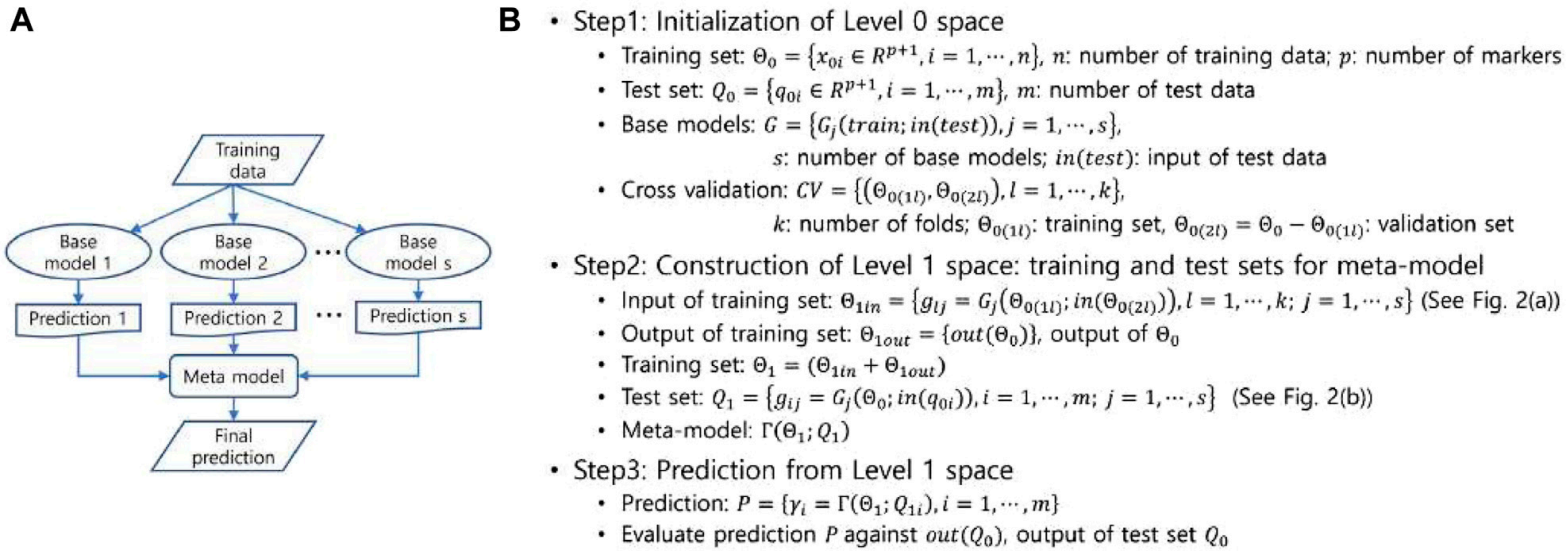
**(A)** A schematic representation of the stacked generalization. **(B)** The decomposed process of the stacked generalization into three steps.

Ensemble methods in machine learning use multiple learning models to improve predictive capabilities beyond what any single model can achieve in isolation. Due to their ability to mitigate overfitting and capture different facets of the data, these methods are widely used and have demonstrated success in various problem domains (Rokach, 2010). Common categories of ensemble methods include bootstrap aggregation (or bagging), boosting, and stacked generation (or stacking) (Nguyen et al., 2021). Broadly speaking, ensemble methods can be classified as parallel or sequential approaches. Parallel methods, such as bagging and stacking, involve the independent training of multiple models, while sequential methods, such as boosting, iteratively train a single model. Parallel methods are further divided into homogeneous and heterogeneous types based on the similarity of the multiple models. In our study, we chose stacking, a heterogeneous parallel method, to take advantage of the diversity inherent in different base models. Stacking differs from other ensemble methods in that it introduces a meta-model in addition to the base models, as shown schematically in Figure 1A. The meta-model is trained using predictions from the base models to generate the final predictions. Stacking usually outperforms the use of a single model (Wolpert, 1992) and applies to both supervised learning (Breiman, 1996b; Ozay and Vural, 2012) and unsupervised learning (Smyth and Wolpert, 1999) tasks.

**FIGURE 2:**
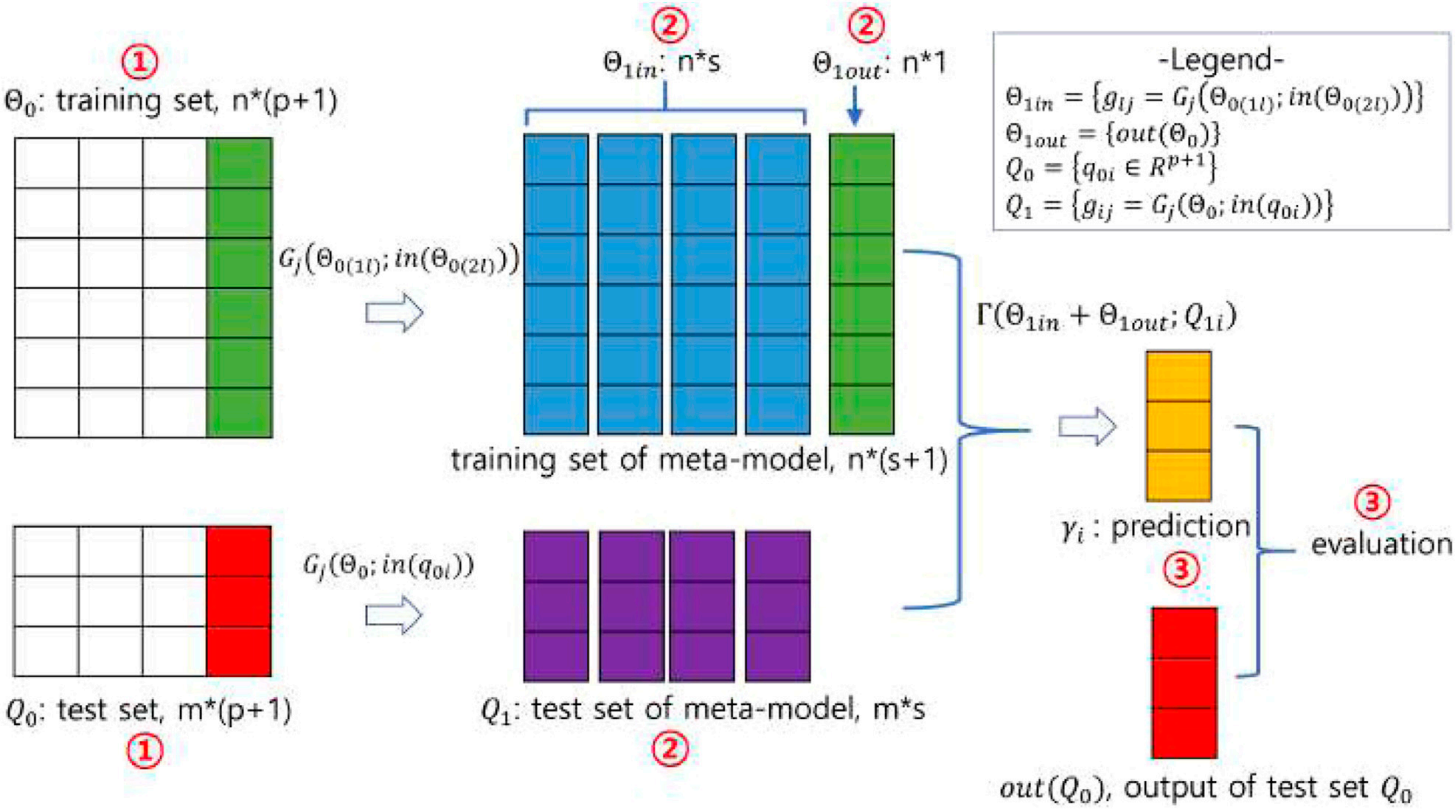
A pictorial demonstration of the stacking procedure. Using the notations in Figure 1B, the number of training and test data is n=6 and m=3 , respectively, the number of dimensions is p=3 , the number of base models is s=4 with 3-fold cross-validation. Note that the circled numbers represent the steps of the stacking procedure shown in Figure 1B and the same data are colored the same.

In stacking, predictions for the data are generated by the base models, and these predictions are then fed into the meta-model, which combines them to produce the final predictions. The stacking process, illustrated in Figure 1B and pictorially exemplified in Figure 2, involves three distinct steps. The first step involves initialization, which includes preparing the training and test data, selecting the base models, and configuring the k -fold cross-validation (CV) (Stone, 1974). As shown in Figures 1B, 2, the training and test sets are represented as n×(p+1) and m×(p+1) matrices, respectively, where n and m denote the number of training and test data, respectively. Additionally, (p+1) represents the dimensionality ( p is the number of SNP markers in our case) of each data point, with a phenotype as output. As a resampling technique, k -fold CV randomly divides the training data into k subsets (or folds) of equal size. A model is trained on (k−1) folds, using the remaining single fold as validation data. This CV process is repeated k times until all k subsets have been used once as validation data, ensuring that all data is used for both training and validation.

**FIGURE 3:**
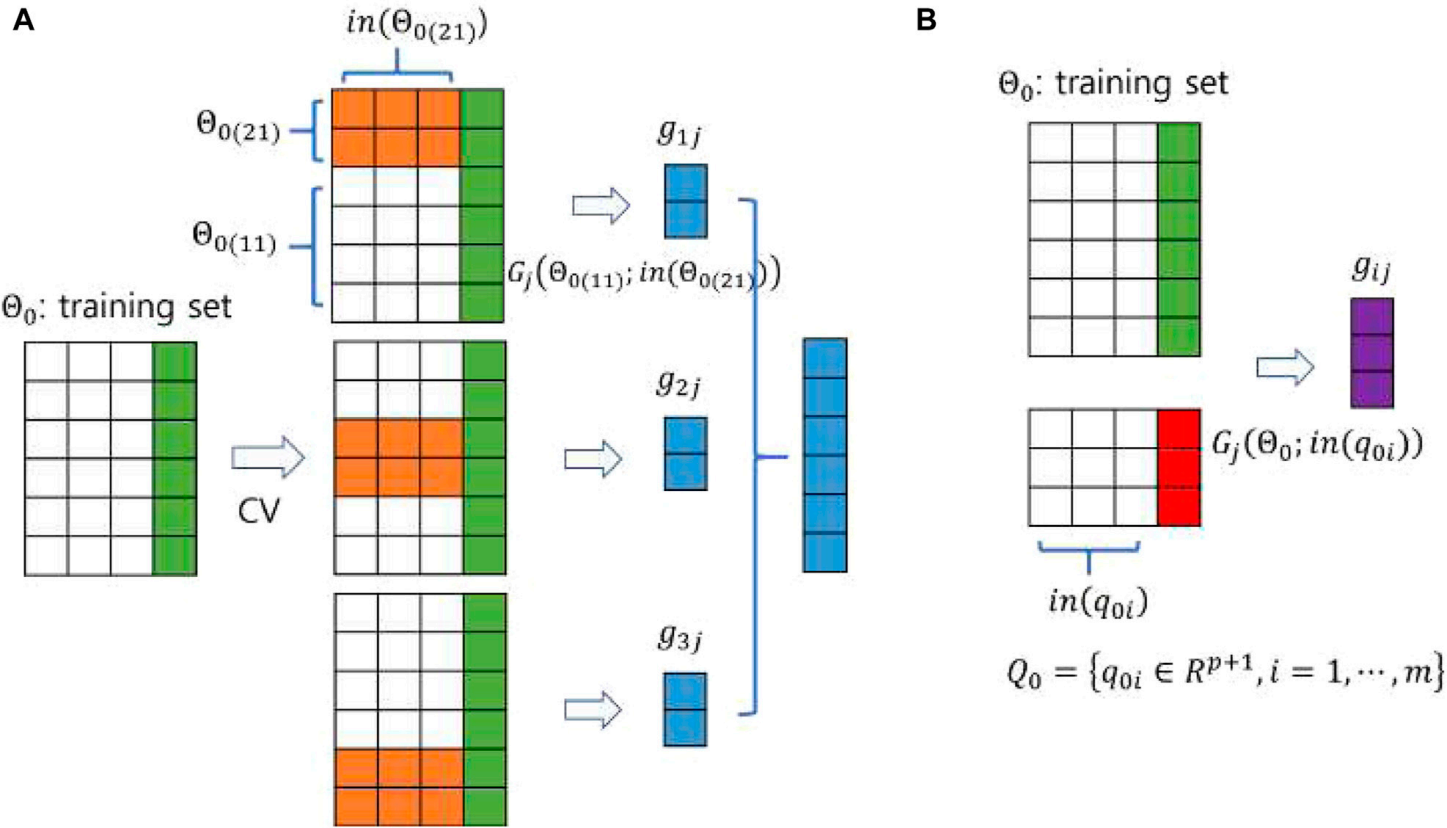
A pictorial demonstration of constructing the input of **(A)** the training set and **(B)** the test set for the meta-model using a 3-fold CV. The sizes of the training and test data are 6×(3+1) and 3×(3+1) , respectively. Note that the same data are colored the same and the notations in Figure 1B are used.

In the second step, the base models are trained on (k−1) folds of the training data while predicting the validation fold. This process results in each base model generating predictions k times, resulting in a total of n predictions, equal to the size of the training samples. These predictions serve as the training data for the meta-model, combined with the phenotype information from the original training data. The test data for the meta-model consists of predictions on the original test data generated by the base models trained on the original training data. Figure 3 illustrates how each base model constructs the input to the meta-model using both training and test data. In this figure, a 3-fold cross-validation was applied to the training data consisting of six instances. Under 3-fold CV, each base model predicts two instances in each iteration, resulting in six predictions, which is equal to the size of the training samples (Figure 3A). Similarly, each base model learns from the original training data and predicts the test data, resulting in three predictions (Figure 3B).

In the third step, the meta-model uses the predictions from the base models as its training data and learns to combine them effectively, generating the final prediction using the test data. The training and test data are constructed in the second step. The meta-model is flexible and can take the form of any type of machine learning model. The prediction produced by the meta-model is evaluated against the output (or phenotype) of the original test data. In the third step shown in Figure 2, the meta-model learns on training data derived from a (4+1)×6 matrix and is then tested on test data derived from a 4×3 matrix generated by four base models to predict three values.

### 2.5 Performance measures

The performance of the proposed stacking model is evaluated against that of each base model through independent learning and prediction of phenotypes. Each model independently learns and predicts phenotypic values based on genetic markers. Both the proposed model and the base models learn from the provided training data and then predict phenotypes using the test data. We then evaluate the performance of the models by comparing the predicted phenotypic values using the proposed model and the base models. We run 20 independent trials to obtain statistical measures for predicted values. Each trial randomly divides the data set into 80% for training and 20% for testing.

We measure model performance using the mean squared error (MSE), which quantifies the average squared difference between observed and predicted values. For the stacking model and each base model, the absolute difference (or error) is calculated on the test data (i=1,2,…,m) as

$$e_{i,stack}\equiv \left|y_{i}^{\mathrm{test}}-{\hat{y}}_{i,stack}\right|\;\;\text{and}\;\;e_{i,base}\equiv \left|y_{i}^{\mathrm{test}}-{\hat{y}}_{i,base}\right|.$$ (3)

Here, $y_{i}^{\mathrm{test}}$ represents the observed value in the test data, ${\hat{y}}_{i,stack}$ and ${\hat{y}}_{i,base}$ denote the predicted values using the proposed model and each base model, respectively. The MSE is then defined as

$${\text{MSE}}_{\mathrm{stack}}^{\mathrm{test}}=\frac{1}{m}\overset{m}{\underset{i=1}{\sum }}e_{i,stack}^{2}\;\;\text{and}\;\;{\text{MSE}}_{\mathrm{base}}^{\mathrm{test}}=\frac{1}{m}\overset{m}{\underset{i=1}{\sum }}e_{i,base}^{2},$$ (4)

where m represents the number of samples in the test data.

In addition to the MSE, overfitting is another critical aspect of performance evaluation. Overfitting, a common problem in machine learning, occurs when the model becomes overly tuned to the training data, hindering its ability to generalize to new data, such as test data. Therefore, one way to measure the extent of overfitting is to compare the prediction errors derived from test and training data. The prediction error from the test data is represented by Eq. 3, while the prediction error from the training data is expressed as

$${\text{MSE}}_{stack}^{train}=\frac{1}{n}\overset{n}{\underset{j=1}{\sum }}{\left|y_{j}^{train}-{\hat{y}}_{j,stack}\right|}^{2}\;\;\text{and}\;\;{\text{MSE}}_{\mathrm{base}}^{train}=\frac{1}{n}\overset{n}{\underset{j=1}{\sum }}{\left|y_{j}^{train}-{\hat{y}}_{j,base}\right|}^{2},$$ (5)

where n is the number of samples in the training data and $y_{j}^{train}$ is the j th sample in the training data. Note that Eq. 5 is used in quantifying overfitting and expresses the mean squared error from the models that are learned and predicted using the same training data. Furthermore, ${\hat{y}}_{j,stack}$ and ${\hat{y}}_{j,base}$ denote the predicted values for the j th training sample predicted by the stacking model and each base model, respectively. It needs to clarify that these predictions are derived from training data, not from test data. In essence, they represent the results when the model is trained and evaluated on the same data sets.

With these settings, the degree of overfitting can be quantified using Eqs 4, 5 as

$$O_{\mathrm{stack}}\equiv \left|{\text{MSE}}_{\mathrm{stack}}^{\mathrm{test}}-{\text{MSE}}_{\mathrm{stack}}^{\mathrm{train}}\right|\;\;\text{and}\;\;O_{\mathrm{base}}\equiv \left|{\text{MSE}}_{\mathrm{base}}^{\mathrm{test}}-{\text{MSE}}_{\mathrm{base}}^{\mathrm{train}}\right|.$$ (6)

$O_{\mathrm{stack}}$ (or $O_{\mathrm{base}}$ ) serves as a metric to measure the fit of the proposed model (or each base model) to the training data. The more the model fits overly to the train data, the smaller ${\text{MSE}}_{\mathrm{stack}}^{\mathrm{train}}$ $(\text{or}{\text{MSE}}_{\mathrm{base}}^{\mathrm{train}})$ . Thus, for similar values of ${\text{MSE}}_{\mathrm{stack}}^{\mathrm{test}}$ and ${\text{MSE}}_{\mathrm{base}}^{\mathrm{test}}$ , a larger discrepancy in the mean squared errors, denoted by $O_{\mathrm{stack}}$ (or $O_{\mathrm{base}}$ ), implies a higher probability of overfitting.

### 2.6 Power of a test and hypothesis test of the non-inferiority

We use a statistical hypothesis test on prediction error to evaluate the performance of the proposed model relative to the base models. Before running any hypothesis test, it is important to confirm that the data meet certain requirements for quantities such as sample size and test power. In our case, the sample sizes of the data from the five species are predetermined. As a result, the sample size becomes a fixed parameter, prompting an initial inquiry as to whether the specified sample size provides adequate test power.

For this purpose, we express the sample size N in terms of sample prediction errors between the set of observed and predicted phenotypes. The relationship is given by (Das et al., 2016).

$$N=\frac{2{\left(Z_{\alpha /2}+Z_{1-\beta }\right)}^{2}S_{p}^{2}}{{\left({\bar{e}}_{\mathrm{base}}-{\bar{e}}_{\mathrm{stack}}\right)}^{2}},\;\;\text{where}\;\;S_{p}^{2}=\frac{S_{\mathrm{base}}^{2}+S_{\mathrm{stack}}^{2}}{2}.$$ (7)

Here, $Z_{\alpha /2}$ and $Z_{1-\beta }$ are the critical values for level α/2 and 1−β , respectively; α and β are type I and type II errors. In addition, ${\bar{e}}_{\mathrm{stack}}$ and $S_{\mathrm{stack}}^{2}$ are the mean and sample variance of $e_{i,stack}$ given in Eq. 3, and similarly for ${\bar{e}}_{\mathrm{base}}$ and $S_{\mathrm{base}}^{2}$ . From Eq. 7, we can evaluate the test power for a given sample size. The test power is formally defined as the probability of correctly rejecting the null hypothesis when it is false. According to Eq. 7, the power, 1−β , expressed in terms of the critical value $Z_{1-\beta }$ is formulated as follows:

$$Z_{1-\beta }=\sqrt{\frac{N}{S_{\mathrm{base}}^{2}+S_{\mathrm{stack}}^{2}}}\left|{\bar{e}}_{\mathrm{base}}-{\bar{e}}_{\mathrm{stack}}\right|-Z_{\alpha /2}.$$ (8)

If power is lower than expected for a given sample size, it is prudent not to use the conventional significance (or superiority) test, but to consider an alternative. This precaution is warranted because reduced power for a given sample size reduces the likelihood of detecting significance if it exists. Moreover, if a new method, such as stacking in our case, offers advantages over existing methods, such as robustness to overfitting, its non-inferiority may still be attractive. In such cases, the non-inferiority test (Schumi and Wittes, 2011; Walker, 2019) provides insight into efficacy, even if it does not establish superiority in terms of efficacy.

Non-inferiority testing is commonly used in medical research, particularly when evaluating new treatments that are expected to outperform existing treatments. In cases where the new treatment offers advantages such as cost-effectiveness and fewer side effects, special statistical tests are used to determine whether the new treatment is no less clinically effective than current standards. These tests, known as non-inferiority trials, determine whether the effect of the new treatment is not significantly worse than existing treatments, taking into account a predefined range known as the non-inferiority margin. This margin represents the maximum difference that is considered clinically acceptable between the effects of the new treatment and existing treatments. Establishing the non-inferiority margin thus becomes a crucial and complicated facet of trial design.

There are several methods for determining the non-inferiority margin, including the synthesis method and the confidence interval method (Althunian et al., 2017). It typically involves weighing several factors, such as clinical relevance, statistical feasibility, expert consensus, and ethical considerations. In the statistical analysis of medical or clinical trials, the required sample size is often calculated based on the Type I error rate and the test power. In our case, however, the sample size is fixed rather than variable, which simplifies the estimation of the non-inferiority margin.

In the non-inferiority test, the relationship between sample size and other parameters, as shown in Eq. 7, is modified including the non-inferiority margin Δ . It is given as (Walker, 2019).

$$N=\frac{2{\left(Z_{\alpha /2}+Z_{1-\beta }\right)}^{2}S_{p}^{2}}{{\left\{\left({\bar{e}}_{\mathrm{base}}-{\bar{e}}_{\mathrm{stack}}\right)-\Delta \right\}}^{2}}.$$ (9)

When α and 1−β are chosen, usually α=0.05 and 1−β=0.8 , we can evaluate the margin Δ from the fixed sample size N given in Eq. 9. That is, the non-inferiority margin is given by, under the restriction of Δ>0 ,

$$\Delta =\left({\bar{e}}_{\mathrm{base}}-{\bar{e}}_{\mathrm{stack}}\right)+\sqrt{\frac{S_{\mathrm{base}}^{2}+S_{\mathrm{stack}}^{2}}{N}}\left(Z_{\alpha /2}+Z_{1-\beta }\right).$$ (10)

For a predetermined sample size N , once α and 1−β are given, we can evaluate the non-inferiority margin.

For comparative performance analysis, we subject the differences in the mean prediction errors between the stacking model and each base model to a statistical hypothesis test. When comparing the means of prediction errors from two different models, the hypothesis of the non-inferiority test is stated as

$$H_{0}:\mu_{\mathrm{base}}-\mu_{\mathrm{stack}}\leq \Delta \;\;\text{and}\;\;H_{1}:\mu_{\mathrm{base}}-\mu_{\mathrm{stack}}>\Delta ,$$ (11)

where $\mu_{\mathrm{base}}$ and $\mu_{\mathrm{stack}}$ are the population means of prediction error for each base model and the stacking model, respectively. Note that the sample prediction error is given in Eq. 3.

**FIGURE 4:**
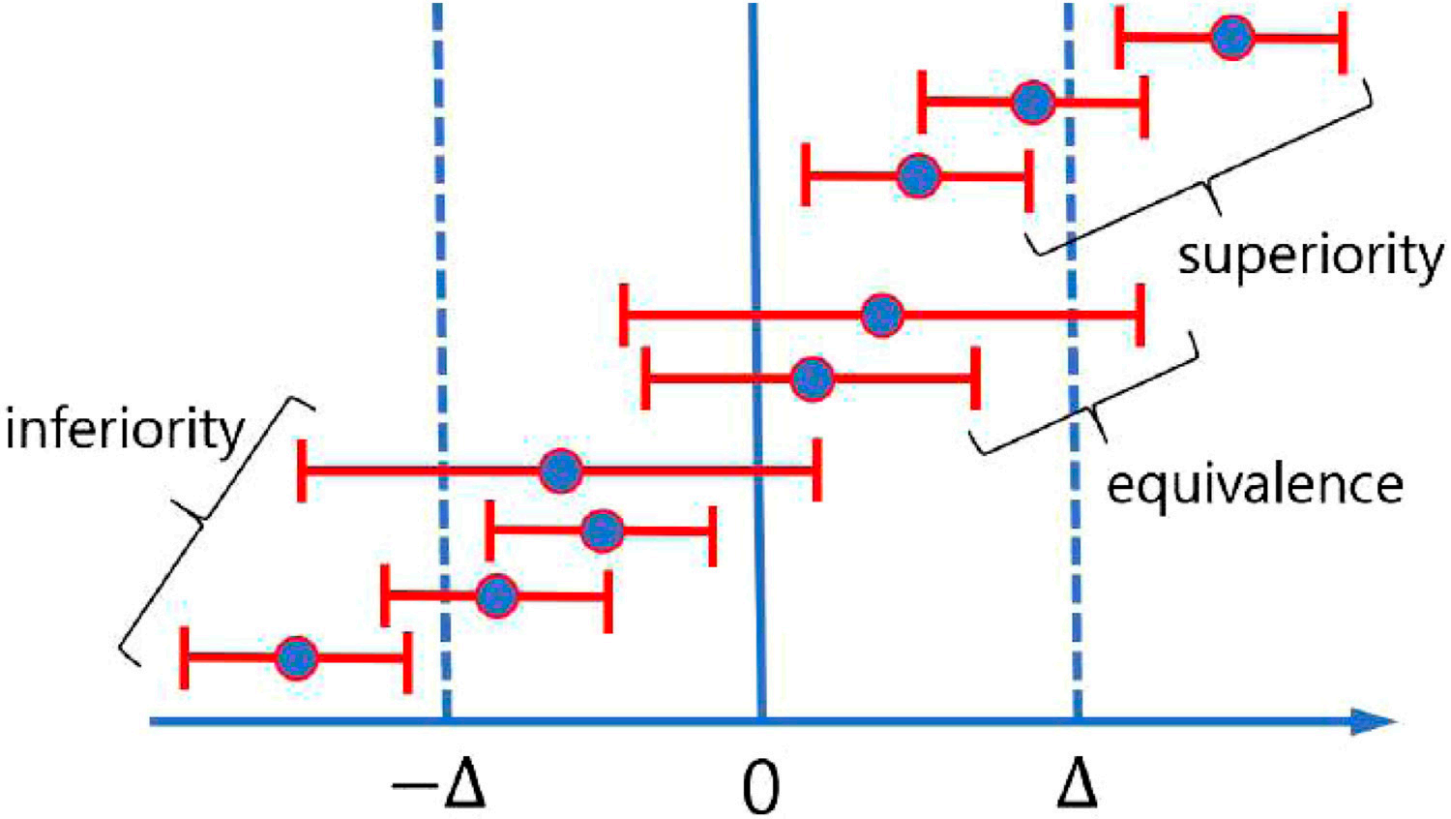
Pictorial demonstration of possible three distinct outcomes of a non-inferiority test using the confidence interval.

The results of non-inferiority tests can be categorized into three distinct outcomes: superiority, non-inferiority (or equivalence), and inferiority (Schumi and Wittes, 2011). Failure to reject the null hypothesis indicates an inferior outcome, whereas rejection may indicate either superiority or equivalence. Superiority is confirmed if the lower bound (LB) of the confidence interval of the mean difference exceeds zero, while equivalence is established if the lower bound exceeds the negative of the predefined margin and the upper bound (UB) exceeds zero (i.e., LB>−Δ and UB>0 ). Inferiority is established if the test result is neither superior nor equivalent. Figure 4 demonstrates the three distinct outcomes of a non-inferiority test. It is worth noting that a superior result is consistent with the conventional notion of statistical significance.

We use the Wilcoxon signed-rank test (Conover, 1999) instead of the t test. This test serves as the nonparametric counterpart to the paired t test. Unlike the t test, which assumes normality, the Wilcoxon test is nonparametric and thus does not require the normality assumption. With the sign function sign(x)=1 if x>0 and sign(x)=−1 if x<0 , the test statistics is the signed-rank sum:

$$T=\overset{m}{\underset{i=1}{\sum }}sign\left(X_{i}\right)R_{i},$$ (12)

where m is the number of data, $X_{i}\equiv e_{i,base}-e_{i,stack}$ from Eq. 3, and $R_{i}$ is the rank of $\left|X_{i}\right|$ in the ascending order. For each base model, this test is performed under the null hypothesis specified in Eq. 11. Running these tests has been facilitated by “wilcox.test” function within the R package “STAT” (Bolar, 2024) using the location shift parameter “mu” set to the margin Δ .

## 3 Results and discussion

We performed the performance comparison for all quantitative traits in the five data sets: rice, barley, maize, mice, and millet. For each species, we ran 20 independent experiments for each phenotype to obtain statistics, such as mean and standard error, of the quantities of interest. In each experiment, we randomly split the data set of each species into 80% training data to learn the models and 20% test data to predict the phenotype. How to split the data set is a hyper-parameter in the sense that the data set does not estimate the split ratio. There is a rule of thumb for how to split a data set into training and test sets. Most studies use an 80/20 or 75/25 split. We used the 80/20 split according to Ref. (Géron, 2023).

### 3.1 Prediction accuracy, overfitting, and test power

**FIGURE 5:**
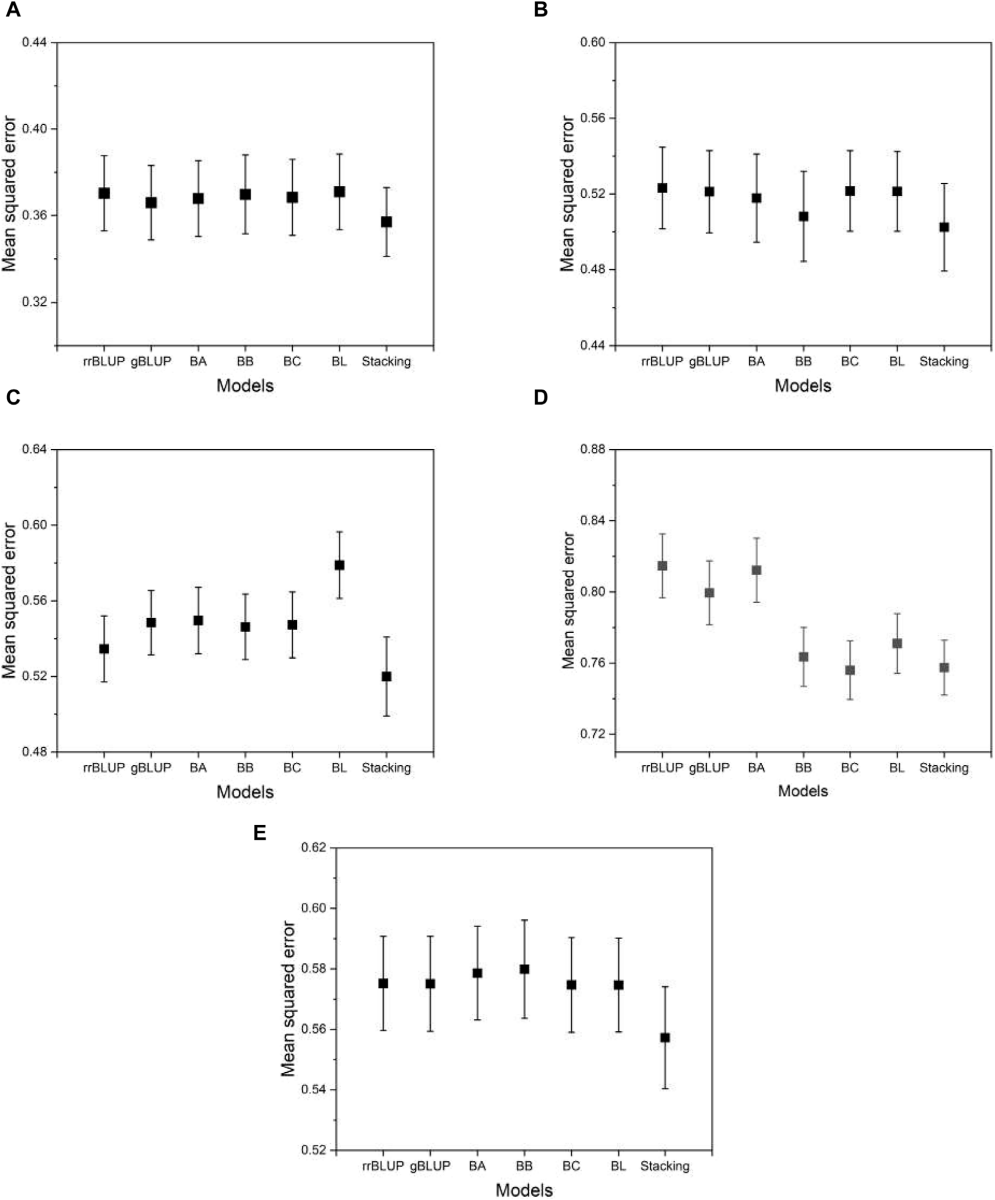
The MSE estimated from the proposed and the base models to compare performance. **(A)** phenotype BRW in the rice data, **(B)** SSW in barley, **(C)** EP in maize, **(D)** BTC in mice, and **(E)** MSPD in millet. Error bars represent standard errors estimated from 20 independent experiments.

Figure 5 shows the typical MSE given in Eq. 4 as the prediction accuracy for the phenotypes evaluated using the proposed model and the base models. From Figure 5, we can see that the MSE evaluated by the proposed model is on average smaller than that of the base models. This means that the proposed model achieves higher, although not significantly higher, prediction accuracy than the base models. This demonstrates the advantage of the proposed model, which integrates the base models to exploit their predictive capabilities. Similar results were found for the other phenotypes in the data set of the five species. The result of the mean squared error for all phenotypes from five species is presented in Supplementary Data S1.

**FIGURE 6:**
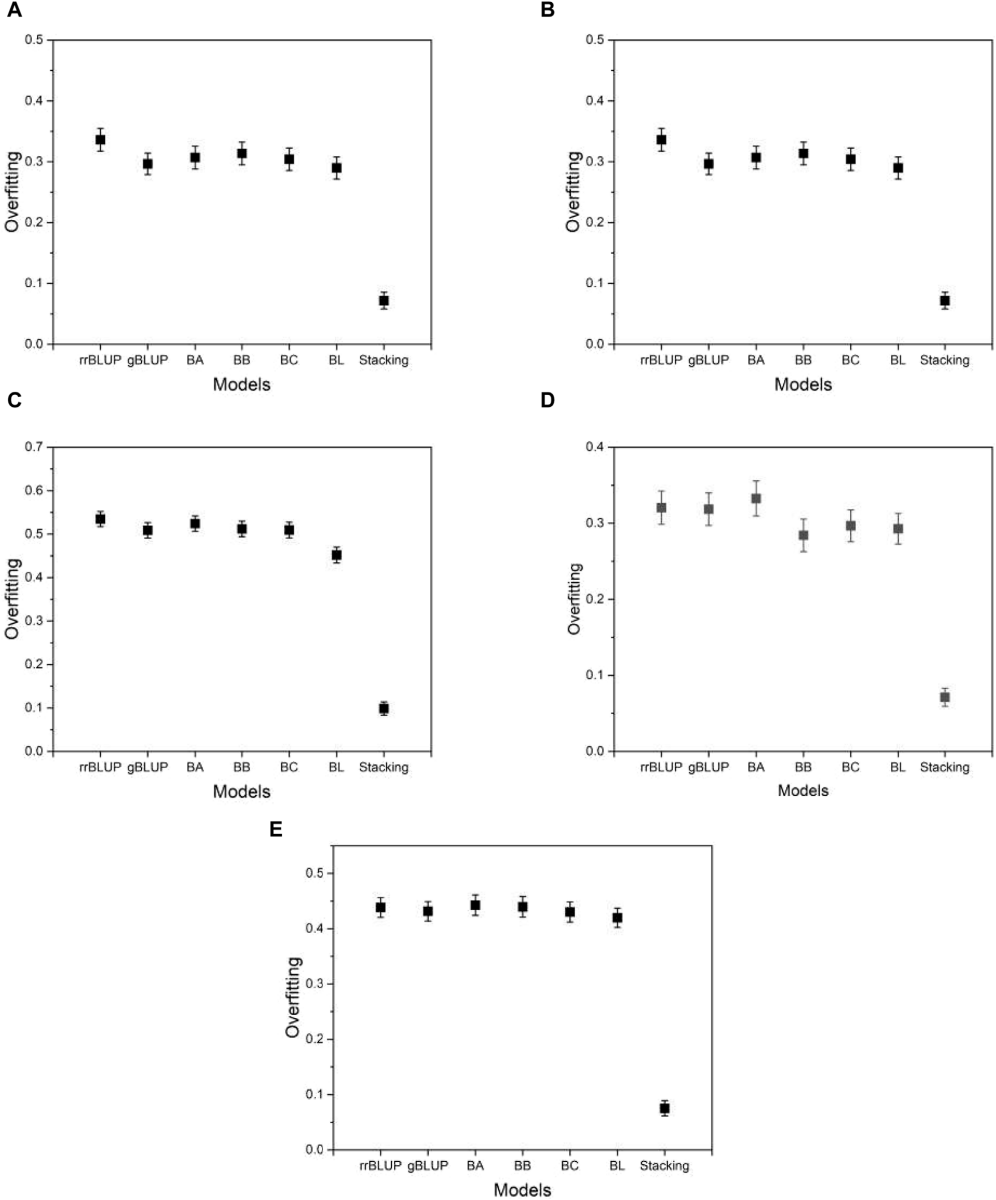
Plots of the overfitting quantified by Eq. 6. **(A)** phenotype BRW in the rice data, **(B)** SSW in barley, **(C)** EP in maize, **(D)** BTC in mice, and **(E)** MSPD in millet.

In addition to the MSE, we measure the degree of overfitting as defined in Eq. 6. Overfitting is a common pitfall in model learning where a model becomes overly tuned to the training data and consequently fails to generalize well to unseen data, such as test data. This phenomenon compromises the intended functionality of the model. As shown in Figure 6, the proposed model exhibits significantly lower levels of overfitting than the base models, while the base models themselves exhibit similar levels of overfitting to each other. This result suggests that the proposed model has an advantage over the base models due to its improved resistance to overfitting. Thus, even in a case where the proposed model performs comparably to the base models, its inherent robustness to overfitting provides a distinct advantage. As Supplementary Data S1 shows, the finding that the proposed model has a higher mean squared error on the training data (not on the test data) than the base models implies that the proposed model fits less accurately than the base models. However, this does not mean that the proposed model is inferior in its performance. The proposed model produced its mean squared error on the test data (not on the training data) lower than that of the base models, as shown in Figure 5. That is, the proposed model predicts the phenotype value more accurately than the base models. The result of the overfitting for all phenotypes from five species is presented in Supplementary Data S2.

Before performing a statistical hypothesis test, we evaluated the power of a test using the data sets of the five species to determine whether the conventional significance test is applicable. If the estimated power (1−β) is sufficiently high (e.g., 1−β≥0.8 ), we can confidently use the conventional significance test. Using Eq. 8, we calculated the power (1−β) for the specified sample sizes in this study, with α=0.05 . The results are detailed in Table 2, in which we find that the power ranges from approximately 0.04–0.07. This indicates that there is a 4%–7% probability that the null hypothesis $\left(H_{0}\right)$ is correctly rejected when the alternative hypothesis $\left(H_{1}\right)$ is true. This result implies that it will be highly unlikely to detect significant test results when there is a notable difference in the performance. Since the sample size is a predetermined quantity, we cannot achieve acceptable power by controlling the sample size. If the power is too low for a given sample size, the test will be unlikely to show significance (or superiority) (Schumi and Wittes, 2011). In such a case, the non-inferiority test can be an alternative to increase the chance of successfully rejecting the null hypothesis when the alternative hypothesis is true. Given these low power values and the advantage of the proposed model over the base models in reducing overfitting, we choose to use the non-inferiority test rather than the conventional significance test.

**TABLE 2:** List of averaged test powers over all phenotypes from five species. The powers were calculated for each base model using Eq. 8. Standard errors are given in parentheses.

Base model	Rice	Barley	Maize	Mice	Millet
rrBLUP	0.037 (0.001)	0.039 (0.002)	0.042 (0.001)	0.074 (0.006)	0.067 (0.011)
gBLUP	0.036 (0.001)	0.039 (0.002)	0.043 (0.002)	0.073 (0.006)	0.055 (0.008)
BA	0.036 (0.001)	0.041 (0.002)	0.043 (0.001)	0.047 (0.002)	0.093 (0.014)
BB	0.036 (0.001)	0.042 (0.002)	0.043 (0.002)	0.039 (0.001)	0.090 (0.013)
BC	0.036 (0.001)	0.039 (0.002)	0.043 (0.001)	0.044 (0.002)	0.089 (0.014)
BL	0.038 (0.001)	0.040 (0.003)	0.053 (0.003)	0.068 (0.005)	0.066 (0.010)

### 3.2 Non-inferiority hypothesis test

Drawing an analogy to establishing the effectiveness of a newly developed treatment in medical or clinical trials (Schumi and Wittes, 2011; Walker, 2019), we used the non-inferiority test to statistically determine whether the proposed stacking model outperforms the current base models. This choice of test is motivated by the observation that there is insufficient power for the conventional significance test and that the proposed model is more resistant to overfitting than the base models. The underlying principle suggests that if the proposed stacking model and the base models demonstrate statistical equivalence in their performance, the stacking method becomes preferable for GS due to its resistance to overfitting in contrast to the base models.

To perform the non-inferiority test, it is necessary to evaluate the test margin Δ given in Eq. 10. Our analysis revealed no significant variance in the margin across the five species, as indicated in Table 3. We examined a hypothesis test regarding the differences in the population means of the prediction errors between the proposed model and each base model, as outlined in Eq. 11. Before running the Wilcoxon signed-rank test, we justified the usage of the Wilcoxon test by testing the normality condition. To this end, we used the Kolmogorov-Smirnov test (Dodge, 2008) under the null hypothesis that mean squared errors follow a normal distribution. If the p value is less than the significance level, the frequency distribution of mean squared errors significantly differs from the normal distribution. We set the significance level at 0.05. We found that a non-negligible fraction of the mean squared errors does not satisfy the normality condition, with the fraction varying between 6% and 66% across the species. Since it is not recommended to use the t test when a test statistic does not follow a normal distribution, we used the Wilcoxon signed-rank test for consistency. Using the Wilcoxon signed-rank test statistic described in Eq. 12, we compared the MSE of the proposed model with that of each base model. Each test involved evaluating the test statistic based on the MSE derived from 20 independent experiments. We present the test results from two perspectives: aggregation across phenotypes and base models.

**TABLE 3:** List of averaged margins over all phenotypes from five species. The margins were calculated for each base model using Eq. 10. Standard errors are given in parentheses.

Base model	Rice	Barley	Maize	Mice	Millet
rrBULUP	0.290 (0.003)	0.240 (0.003)	0.250 (0.005)	0.153 (0.001)	0.178 (0.005)
gBLUP	0.289 (0.003)	0.240 (0.003)	0.258 (0.004)	0.153 (0.001)	0.181 (0.005)
BA	0.287 (0.003)	0.234 (0.003)	0.258 (0.004)	0.147 (0.001)	0.212 (0.007)
BB	0.287 (0.003)	0.230 (0.003)	0.258 (0.004)	0.143 (0.001)	0.223 (0.010)
BC	0.288 (0.003)	0.240 (0.003)	0.258 (0.004)	0.145 (0.001)	0.219 (0.010)
BL	0.293 (0.003)	0.243 (0.003)	0.271 (0.004)	0.151 (0.001)	0.184 (0.005)

**FIGURE 7:**
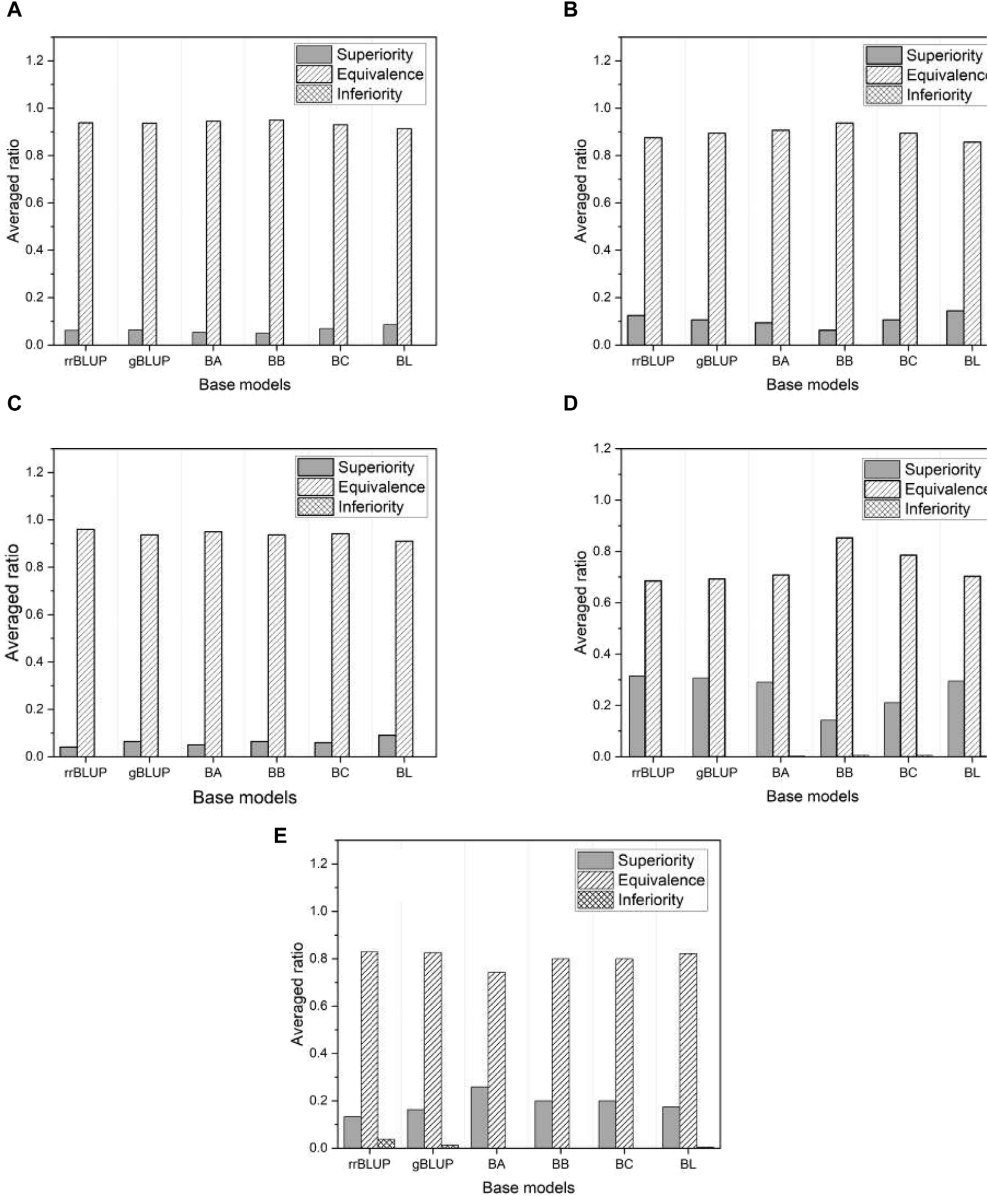
The ratio of three distinct test results averaged over all phenotypes in each of five species: **(A)** rice, **(B)** barley, **(C)** maize, **(D)** mice, and **(E)** millet.

Figure 7 shows the proportion of three different categories (superiority, equivalence, and inferiority) of test results averaged over all phenotypes in each of the five species. As shown in Figure 7, the inferiority result is not observed, indicating that the proposed model is either superior or at least equivalent to all base models. Specifically, we obtained about a 10%–30% superiority result for mice and millet, while about a 5%–10% superiority result for the other species. The performance of the proposed model is further highlighted from an alternative perspective.

**FIGURE 8:**
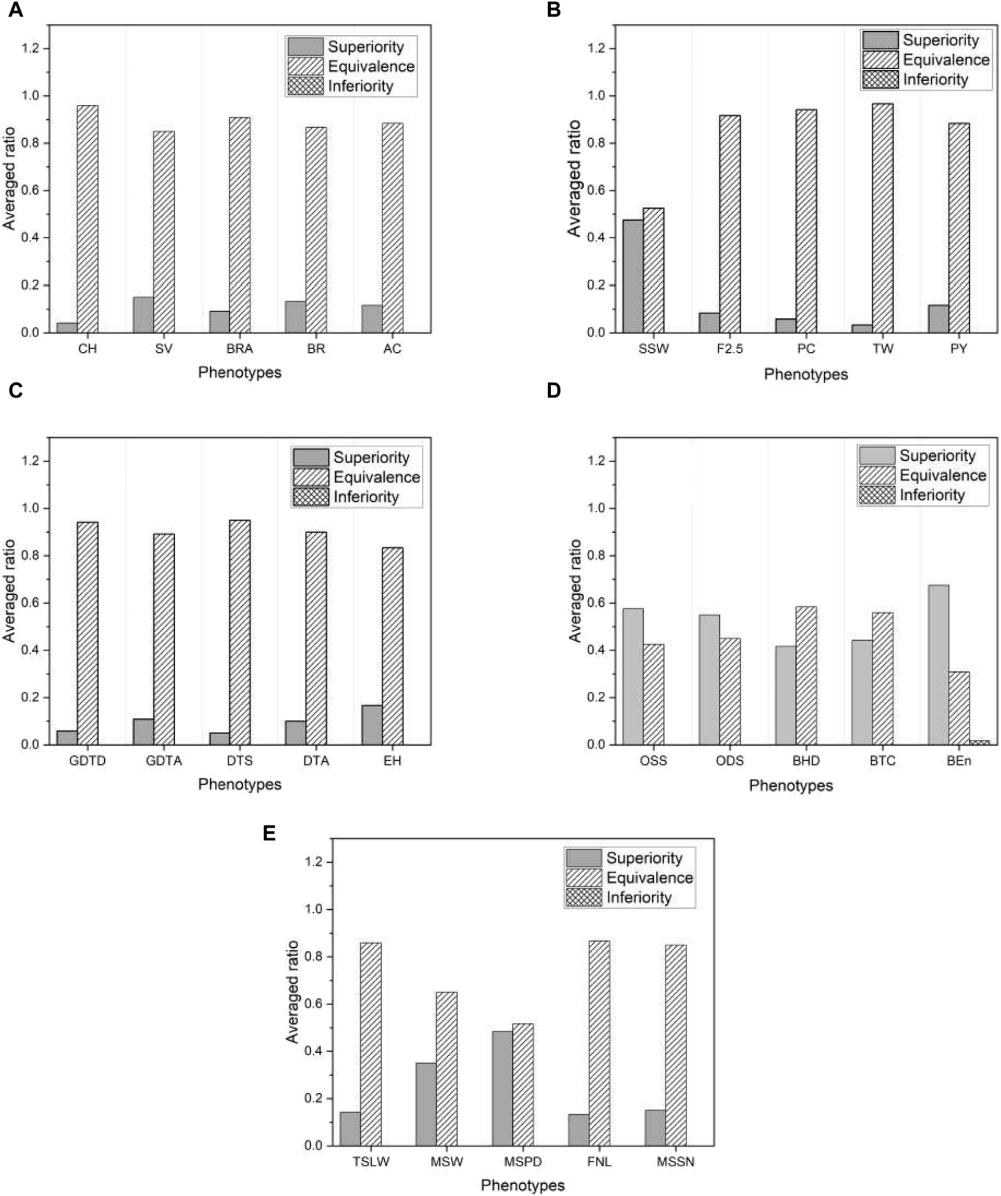
The ratio of three different test results averaged over six base models in five species: **(A)** rice, **(B)** barley, **(C)** maize, **(D)** mice, and **(E)** millet. For visual purposes, the results of five phenotypes are selected for each species.

Figure 8 shows the proportion of the three categories of test results within each selected phenotype, averaged across all base models. Once again, we find that the proposed model outperforms the base models, as there are no inferior results except for a negligible proportion in the phenotype “BEn” of the mice shown in Figure 8D. Note that the proportion of superior results for mice and millet is much higher than for the other species. This is consistent with the higher proportion of superior results for mice and millet than for the other species shown in Figure 7. We further investigate this feature in Section 3.4. Since the proposed model effectively mitigates overfitting, it has an advantage over the base models. The result of the non-inferiority test for all phenotypes from five species is presented in Supplementary Data S3.

**FIGURE 9:**
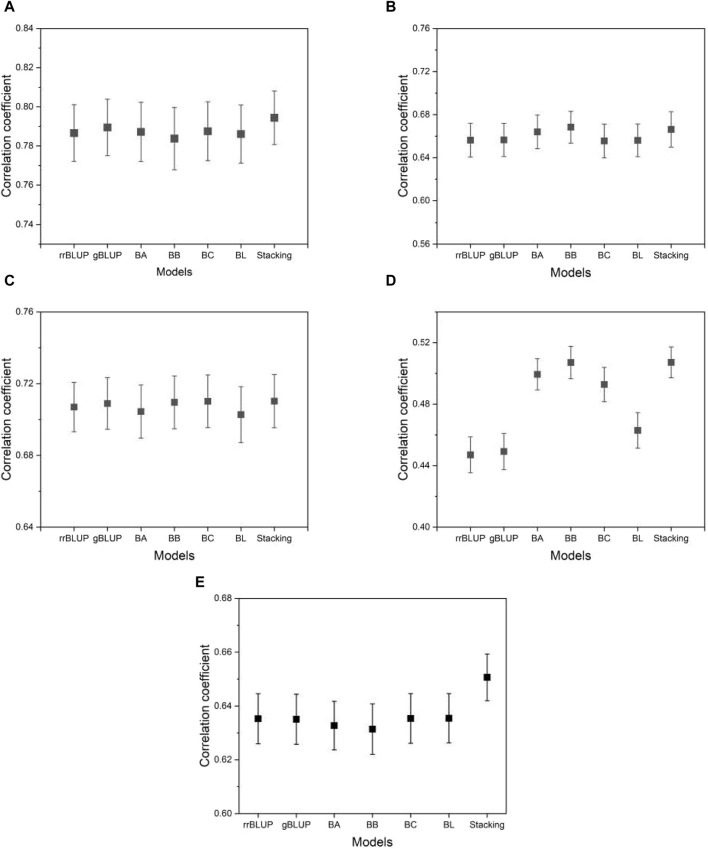
A plot of the correlation coefficients from the proposed model and the base models. **(A)** phenotype BRW in the rice data, **(B)** SSW in barley, **(C)** EP in maize, **(D)** BTC in mice, and **(E)** MSPD in millet. The list of phenotypes is the same as used in Figure 5. Error bars represent standard errors estimated from 20 independent experiments.

In breeding, the MSE may not be the most appropriate metric because it can be affected by constant or scaling factors in the models, potentially inflating the MSE without changing the predictive ranking. As a result, breeders often choose to evaluate predictive accuracy using correlation analysis. This evaluation involves measuring the correlation between the predicted and observed values of individuals in the test data set. A higher correlation indicates better predictive performance. In our research, we use Pearson’s correlation coefficient, a widely used metric in the field of GS, to quantify this accuracy. Figure 9 shows the correlation coefficients estimated from the proposed model and the base models. As we can see from the figure, the proposed model shows a higher or comparable correlation between the predicted and observed phenotypes than the base model. The result of the correlation coefficients for all phenotypes from five species is presented in Supplementary Data S4.

### 3.3 Performance comparison with RKHS and bagging regressor

**FIGURE 10:**
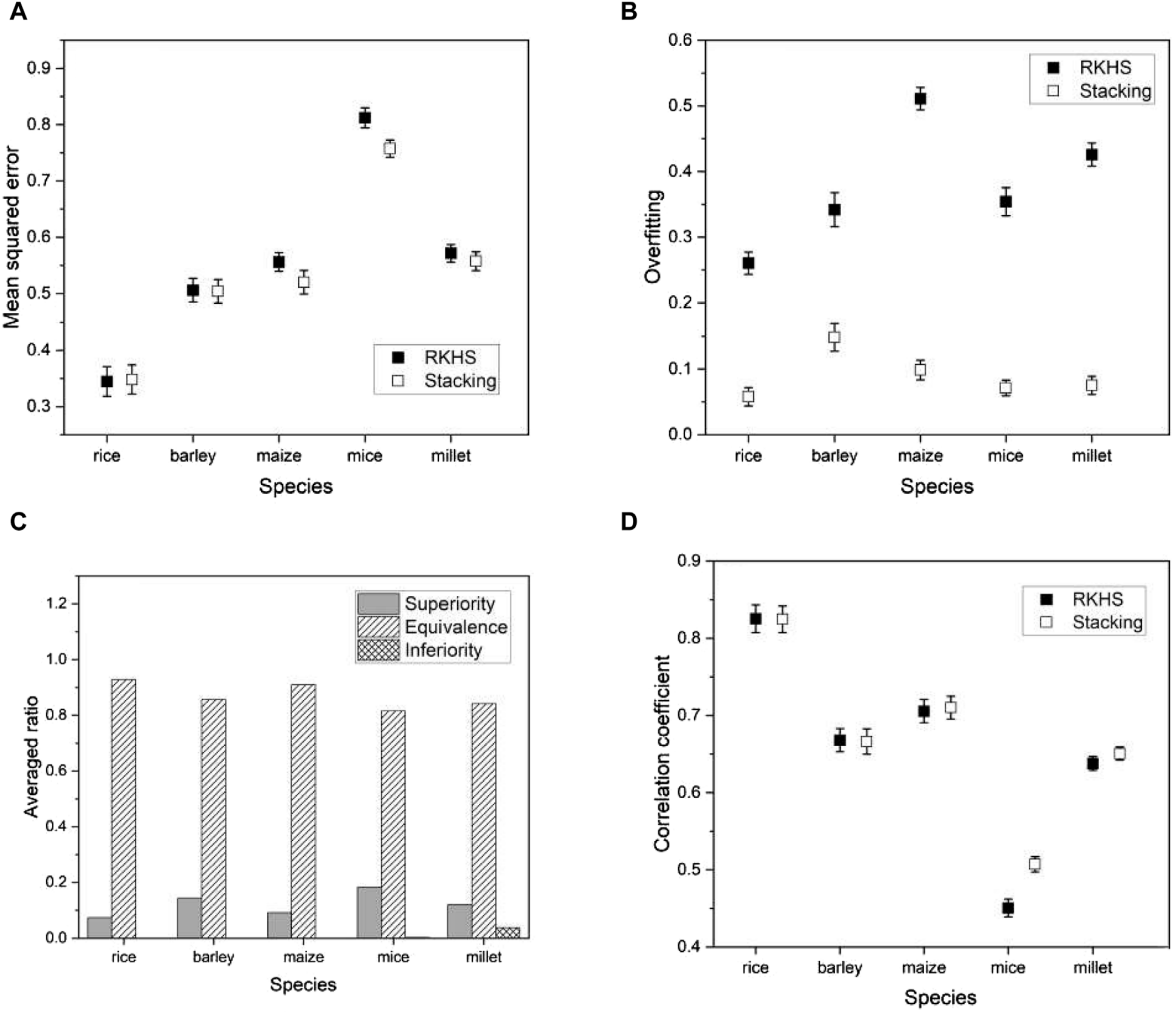
Plots of various performance measures from a phenotype in each species using RKHS (■) and stacking (□) . **(A)** mean squared error, **(B)** overfitting, **(C)** test results, and **(D)** correlation coefficient. The list of phenotypes is the same as used in Figure 5. Error bars represent standard errors estimated from 20 independent experiments.

We introduce a recent genomic selection technique known as reproducing kernel Hilbert space (RKHS) (Montesinos-López et al., 2021) to support the advantages of the proposed model. The core concept of RKHS involves mapping the independent variables (in our context, genotype values) into a theoretically infinite-dimensional Hilbert space using a kernel function. This transformation allows the application of traditional machine learning methods to improve the results. RKHS has gained attraction for its effectiveness in uncovering nonlinear patterns in data sets. We applied RKHS to the same species as before and compared its performance with our proposed model. Evaluation metrics remained consistent and included mean squared error (MSE), degree of overfitting, and Pearson’s correlation coefficient. We conducted a non-inferiority test to determine whether the prediction errors of our proposed model compared favorably with RKHS. Formally, we hypothesized:

$$H_{0}:\mu_{\mathrm{rkhs}}-\mu_{\mathrm{stack}}\leq \Delta \;\;\text{and}\;\;H_{1}:\mu_{\mathrm{rkhs}}-\mu_{\mathrm{stack}}>\Delta ,$$ (13)

where $\mu_{\mathrm{rkhs}}$ and $\mu_{\mathrm{stack}}$ are the population means of the prediction error for RKHS and the stacking model, respectively. The results from a phenotype in each species are shown in Figure 10. We found that the proposed model outperformed RKHS in all evaluation metrics. The results using all phenotypes are given in Supplementary Data S5.

As another ensemble method for comparison with the proposed stacking, we consider a bagging regressor (Breiman, 1996a). Ensemble techniques are commonly divided into three categories: bootstrap aggregation (or bagging), boosting, and stacking. Boosting involves training a single model iteratively, while bagging and stacking involve running multiple models simultaneously. A bagging regressor serves as an ensemble estimator by training multiple models on the original data set and then combining their predictions by averaging to obtain a final prediction. This method often reduces variance by introducing randomness during construction and using ensemble techniques. The primary difference between the proposed stacking and the bagging regressor lies in their approach to using model predictions. Stacking introduces a meta-model and trains a meta-model using predictions from the multiple models (or base models), while the bagging regressor aggregates predictions from the multiple models. For a fair comparison, we used the same multiple models for the bagging regressor as the proposed method.

**FIGURE 11:**
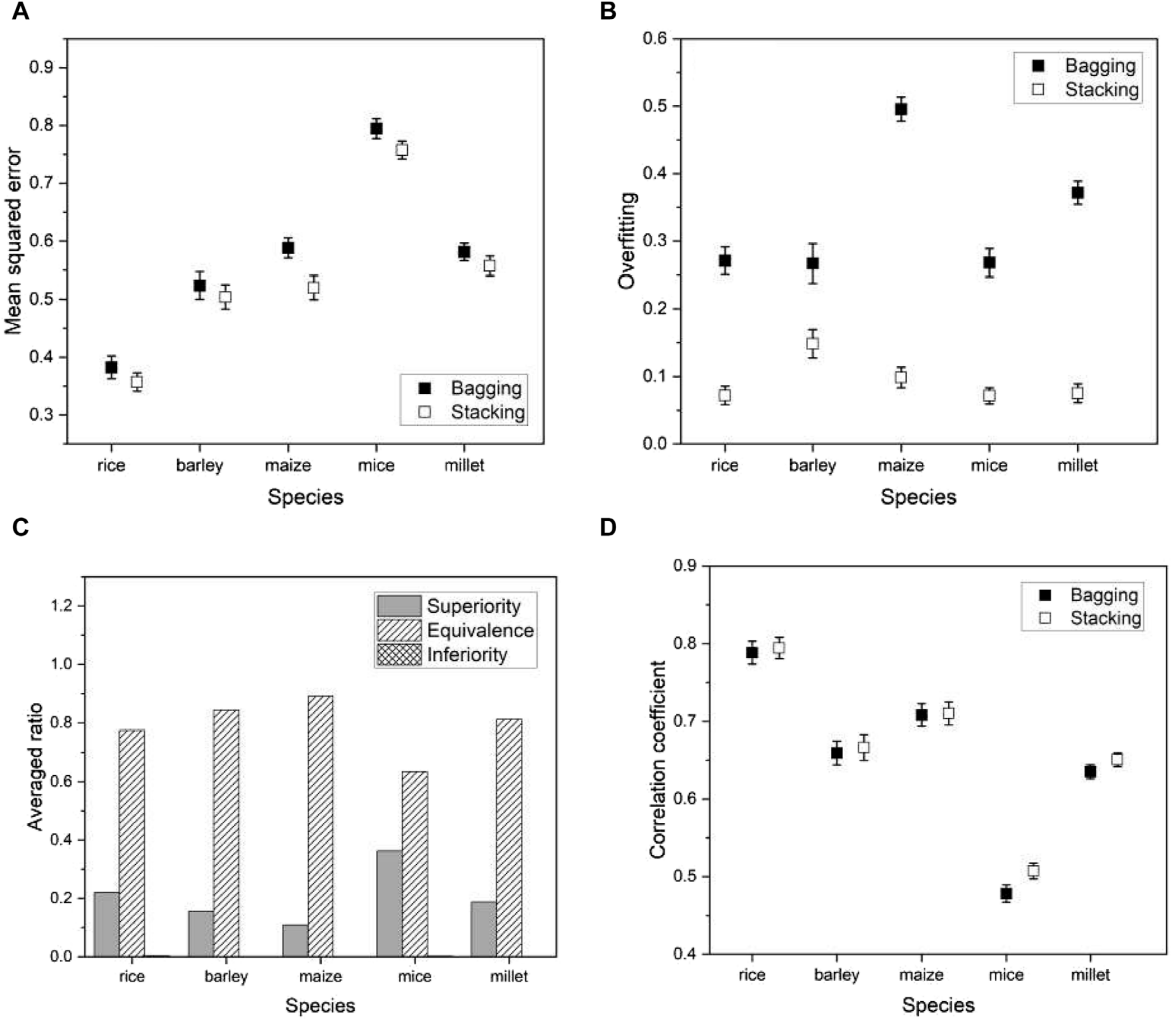
Plots of various performance measures from a phenotype in each species using bagging regressor (■) and stacking (□) . **(A)** the mean squared errors, **(B)** the degree of overfitting, **(C)** the hypothesis testing results for the phenotypes, and **(D)** the correlation coefficients. The list of phenotypes is the same as used in Figure 5. Error bars represent standard errors estimated from 20 independent experiments.

We applied a bagging regressor to the same species as before and compared its performance with the proposed model. Evaluation metrics remained consistent and included mean squared error (MSE), degree of overfitting, and Pearson’s correlation coefficient. We also conducted a non-inferiority hypothesis test to determine whether the prediction errors of our proposed model compared favorably with the bagging regressor. Formally, we hypothesized:

$$H_{0}:\mu_{\mathrm{bagg}}-\mu_{\mathrm{stack}}\leq \Delta \;\;\text{and}\;\;H_{1}:\mu_{\mathrm{bagg}}-\mu_{\mathrm{stack}}>\Delta ,$$ (14)

where $\mu_{\mathrm{bagg}}$ and $\mu_{\mathrm{stack}}$ are the population means of the prediction error for the bagging regressor and the stacking, respectively. We ran the bagging regressor and compared its performance with the stacking. The results using a phenotype in each species and the hypothesis tests of Eqs 13, 14 are shown in Figure 11. We found that the proposed model outperformed the bagging regressor in all evaluation metrics. The results using all phenotypes are given in Supplementary Data S6.

### 3.4 Computation time analysis and effect of allele frequencies on the models

As an ensemble method, the proposed model typically requires more computational resources than individual models. Consequently, the trade-off for achieving improved performance can be articulated in terms of the time complexity of the computation. Time complexity quantifies the computation time required for a method to execute based on the given input. The primary factor contributing to computation time is the training of the models. In stacking, the meta-model is trained using predictions from the base models. These base models, in turn, undergo training and prediction using the cross-validation (CV) technique. As a resampling method, k -fold CV randomly divides the training data into k subsets (or folds) of equal size. Each base model is trained on (k−1) folds, with one fold reserved for validation data during prediction. This CV process is iterated k times until all subsets have been used once for validation, ensuring comprehensive use of data for both training and prediction. Consequently, in a k -fold CV, each base model is trained k times. This implies that for s base models, the additional computation time scales proportionally with the product of k×s . For example, in our case, using 6 base models with 5-fold cross-validation requires about 30 times more computation time for the proposed model compared to each base model individually.

To measure the computation time for each model, we used an R function, “Sys.time,” which provides an absolute time value. The specification of the computational resource we used is as follows:

• CPU: Intel Xeon E5-2695 v4 @ 2.10 GHz (36Core x2)

• RAM: 264 GB

• OS: CentOS 7 × 64

Table 4 presents the computation time (in seconds) for both the proposed model and the base model, averaged over all phenotypes within each species. Note that the computation time for the base model is averaged quantity over six base models. The results from the table show that the proposed method generally requires about 30 times more computation time compared to the base model. Consequently, the proposed model requires more computational resources compared to the base models. Note that given the prevailing capabilities of computing systems, the incremental computational time required is a minimal obstacle in practical applications.

**TABLE 4:** Computation times in seconds, averaged over all phenotypes in each species, with standard errors in parentheses for the proposed and the base models. Note that the computation time for the base model is averaged over all base models.

	Rice	Barley	Maize	Mice	Millet
Base model	7 (3)	10 (4)	85 (38)	58 (24)	57 (23)
Stacking	245 (12)	342 (7)	2,599 (62)	1,651 (23)	1,673 (28)

In GS, three different genotypes are typically represented numerically as −1, 0, 1 (or 0, 1, 2) for recessive homozygous (aa), heterozygous (Aa), and dominant homozygous (AA) genotypes, respectively. In cases where the allele frequencies of three different genotypes do not exhibit linear proportionality with their corresponding phenotype values, there is a possibility that the base model, whether it is a linear mixed model or a Bayesian model, will not perform optimally. The reason is that the base model is essentially a multiple linear regression model that assumes linearity between phenotype and genotype values. Consequently, if the linearity assumption is violated, the base models are unlikely to produce accurate results.

To investigate whether the data used in this study exhibit the linearity, we evaluated the proportions of each genotype present in the data. Table 5 shows the results, revealing three different types of genotype frequencies. In rice and barley, the allele frequencies are predominantly composed of two homozygous genotypes, with the heterozygous genotype being insignificantly represented. In maize, on the other hand, recessive homozygous and heterozygous genotypes predominate, while the dominant homozygous genotype is minimal. In both cases, with effectively two predominant genotype values, the assumption of linearity is inherently satisfied regardless of the phenotype values. In the case of mice, however, all three genotypes have non-negligible allele frequencies. Therefore, it is necessary to examine the extent to which the three genotypes in the mouse data are linearly related to their corresponding phenotypic values.

**TABLE 5:** The averaged ratio of each genotype value over all SNPs and phenotypes from five species. Standard errors are given in parentheses.

Genotype	Rice	Barley	Maize	Mice	Millet
−1	0.811 (0.002)	0.866 (0.002)	0.779 (0.001)	0.445 (0.003)	0.966 (0.001)
0	0.001 (0.001)	0.013 (0.001)	0.204 (0.001)	0.363 (0.001)	0.016 (0.001)
1	0.188 (0.002)	0.121 (0.002)	0.018 (0.001)	0.193 (0.002)	0.018 (0.001)

**FIGURE 12:**
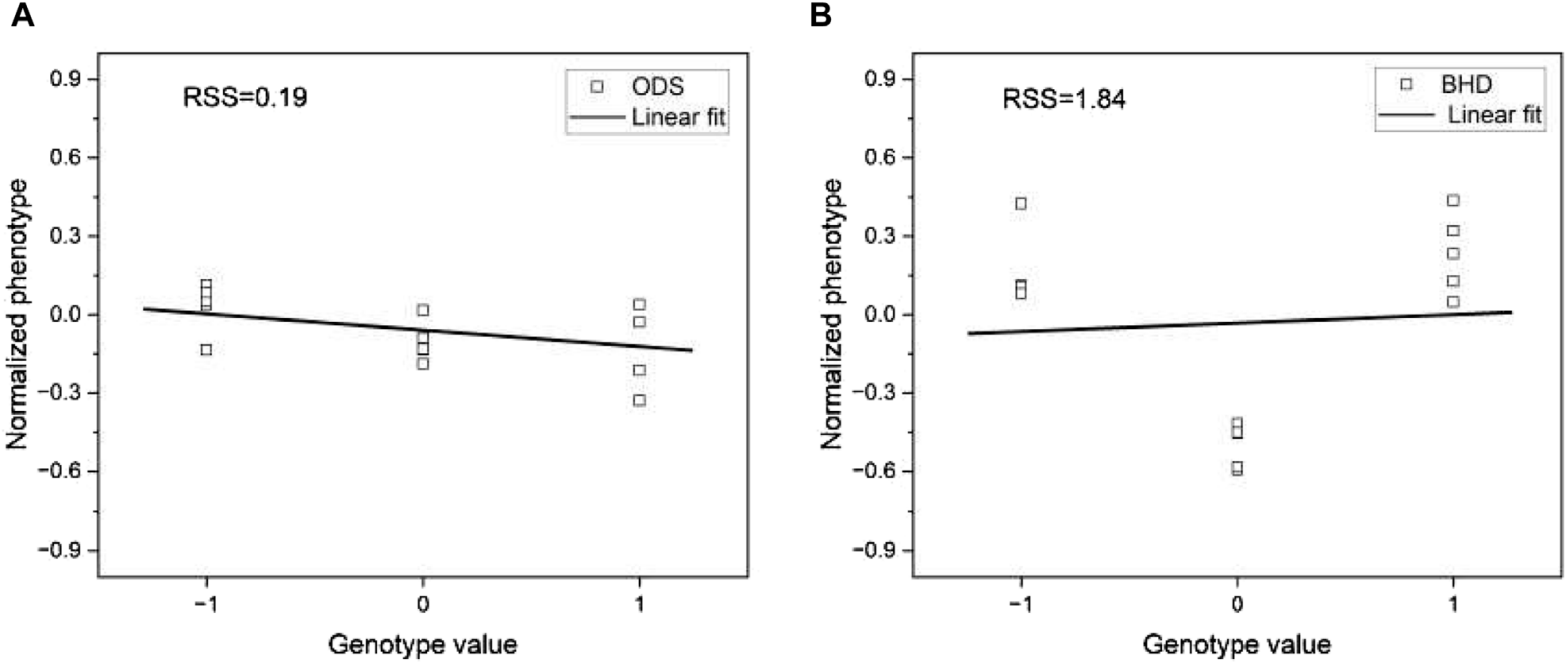
Plots of genotype values *versus* phenotype value for **(A)** phenotype ODS and **(B)** phenotype BHD. The phenotype values for each trait are normalized to compare them in the same range. The lines are the linear fit to the corresponding data.

To accomplish this task, we selected two phenotypes, ODS and BHD, as examples from the mouse data. We then randomly selected one SNP and divided the corresponding genotype and phenotype pairs of the mouse samples into three groups of different genotype values. We also calculated the average phenotype values within each genotype group. In this way, we had an average phenotype for each of the three different genotypes. This process was repeated for five randomly selected SNPs. This allowed us to generate plots showing the averaged phenotypes corresponding to each genotype value, as shown in Figure 12. To ensure a fair comparison in the linear regression, we normalized the phenotype values within each phenotype.

We applied a linear regression model to the pre-processed data and visualized the result in Figure 12. Visual inspection of Figure 12 shows that the fit of the linear regression model varies significantly between the two phenotypes. To quantify the degree of fit, we measured the residual sum of squares (RSS), a metric that quantifies the variance in the residuals of a linear regression model. RSS serves as an indicator of the dissimilarity between the data and an estimated model, with smaller values indicating a better fit. Notably, we observed that the phenotype ODS has a significantly lower RSS of 0.19 compared to the phenotype BHD, whose RSS of 1.84 is approximately ten times that of the phenotype ODS. This result suggests that the higher the RSS, the worse the phenotype prediction of the base models would be.

When the phenotypes of the mouse data lack their linearity (or high RSS), the base models are less accurate in their predictions than in the case of other species. The proposed stacking model, which uses predictions from these base models, is robust to overfitting and exploits the strength of each prediction by leveraging the collective knowledge of multiple models. These features of the proposed model mitigate the imprecision of the base models. To support this claim, we performed the same linear regression fit with all phenotypes and presented the result in Table 6. From Table 6, we can see that as RSS increases, there is a strong tendency for a corresponding increase in the prevalence of superior results. That is, the greater the deviation from linearity, the less accurate the base model. This explains why there is a notable preponderance of superior test results in the mouse data compared to data from other species, as shown in Figures 7, 8.

**TABLE 6:** The list of RSS and the proportions of the three test results of all phenotypes. The result is sorted by RSS in descending order.

Pheno.	RSS	Superior	Equiv	Inferior	Pheno.	RSS	Superior	Equiv	Inferior
OEn	2.31	0.70	0.27	0.03	OBo	0.26	0.06	0.94	0.00
BEn	2.31	0.68	0.31	0.02	BGl	0.20	0.00	1.00	0.00
BHD	1.84	0.42	0.58	0.00	BAL	0.20	0.13	0.87	0.00
BTC	1.40	0.44	0.56	0.00	OSS	0.19	0.58	0.43	0.00
OBM	1.23	0.22	0.78	0.00	ODS	0.19	0.55	0.45	0.00
BSo	0.40	0.18	0.82	0.00	BAS	0.14	0.08	0.92	0.00
BTP	0.38	0.15	0.85	0.00	BLT	0.13	0.15	0.85	0.00
BLD	0.37	0.02	0.98	0.00	BCa	0.12	0.23	0.77	0.00
BAl	0.36	0.08	0.93	0.00	BAg	0.08	0.32	0.68	0.00
BChl	0.30	0.20	0.80	0.00	BUr	0.06	0.03	0.98	0.00

## 4 Conclusion

In this study, we proposed a stacking model as a computational method for genome prediction. The proposed model belongs to the ensemble methods in machine learning and takes a different approach from conventional methods. It integrates base models to explore the collective knowledge from them and uses the meta-model to achieve better performance. Using the data sets with different phenotypes of various species, we demonstrated the advantage of the proposed method by comparing the performance of the proposed model with that of individual base models. We achieved better performance than base models can produce with the proposed method.

In addition to better prediction accuracy compared to base models, the proposed model has other advantages that can make it effective in improving performance. By combining multiple base models, the proposed model learns from diverse predictions, resulting in a reduction of overfitting. This results in more accurate predictions compared to base models and makes it suitable for real-world applications. The proposed model was also less sensitive to the choice of hyper-parameters of the base models, meaning that the default hyper-parameter setting works in many cases. As an ensemble method, the proposed model is robust because it is less affected by the variety of data and prediction tasks than the base models.

It should be noted that the proposed model, as an ensemble method, typically requires more computation and is more complex to implement and tune than base models. However, since the computational complexity of the proposed model generally increases in proportion to the number of base models, the additional computational time required is hardly an obstacle in practice. While the linear base models used in this study can estimate the marker effects, the proposed method does not provide the marker effects. This is because the meta-model predicts the phenotype value, not the marker effects, from the output (i.e., predicted phenotypes) of the base models. However, the marker effects can be estimated indirectly, for example, by taking the weighted average of the estimates from each base model.

In addition to the methodological advantage in prediction, the proposed stacking method has other potential advantages in breeding. Stacking allows breeders to combine desirable traits from multiple parents into a single offspring. This results in offspring that inherit a variety of beneficial traits, such as disease resistance, high yield potential, and improved quality. Stacking also allows breeders to achieve desired traits more quickly by combining the strengths of multiple parents in each generation. This accelerates the breeding process, resulting in faster development of new varieties or breeds with improved traits. By creating plants or animals with improved traits such as disease resistance and environmental adaptability, stacking contributes to the sustainability and resilience of agricultural systems. Overall, stacking allows breeders to speed up the breeding process by efficiently combining desirable traits from different genetic sources, resulting in offspring with improved performance, adaptability, and market value.

In this study, we compared the proposed model with the linear mixed and Bayesian models and did not consider the nonlinear models, such as support vector machines and deep learning. Although the nonlinear models have difficulties in interpreting the results, such as the genetic effects of single markers, these models may outperform linear models, especially when dealing with highly complex, nonlinear relationships between genotypes and phenotypes. We also limited this study to single-trait genomic selection. However, multi-trait genomic selection is useful in various situations where we need to simultaneously improve the performance of more than one trait in a breeding program. Therefore, it would be necessary and important to investigate how the proposed model works for the nonlinear base models and multi-trait GS. The performance of the proposed model may depend on the choice of base models. How the number and type of base models affect the performance of the proposed model also needs to be investigated. These should be understood in future work.

## Data Availability

The original contributions presented in the study are included in the article/Supplementary Material, further inquiries can be directed to the corresponding authors.
