# Supplementary material for: Stacked generalization as a computational method for the genomic selection
Source: Front Genet. 2024 Jul 10;15:1401470. doi: 10.3389/fgene.2024.1401470 (PMC11266134; doi:10.3389/fgene.2024.1401470)
Supplement: Supplementary file 1 [file DataSheet1.zip › supplementary_material_updated.pdf]

Supplementary Material

1 SUPPLEMENTARY TABLES

S1 Table

No	Abbreviation	Phenotype	No	Abbreviation	Phenotype
1	FTAR	Flowering time at Arkansas	16	SL	Seed length
2	FTF	Flowering time at Faridpur	17	SW	Seed width
3	FTAB	Flowering time at Aberdeen	18	SV	Seed volume
4	FTRA	FT ratio of Arkansas Aberdeen	19	SS	Seed surface area
5	FTRF	FT ratio of Faridpur Aberdeen	20	BRL	Brown rice seed length
6	CH	Culm habit	21	BRW	Brown rice seed width
7	FLL	Flag leaf length	22	BRA	Brown rice surface area
8	FLW	Flag leaf width	23	BRV	Brown rice volume
9	PN	Panicle number per plant	24	SLW	Seed length width ratio
10	PH	Plant height	25	BRR	Brown rice length width ratio
11	PL	Panicle length	26	SHS	Straight head susceptibility
12	PP	Primary panicle branch number	27	BR	Blast resistance
13	SN	Seed number per panicle	28	AC	Amylose content
14	FP	Florets per panicle	29	ASV	Alkali spreading value
15	PF	Panicle fertility	30	PC	Protein content

Table S1. A list of 30 quantitative traits in the rice data used in this study.

S2 Table

No	Abbreviation	Phenotype
1	SSW	Standardized seed weight
2	F2.2	Weight of seeds with the size between 2.2 and 2.5
3	F2.5	Weight of seeds with the size between 2.5 and 2.8
4	F2.8	Weight of seeds with the size greater than 2.8
5	PC	Protein content
6	TW	Test weight
7	EC	Ergosterol content
8	PY	Protein yield

Table S2. A list of seven quantitative traits in the barley data used in this study.

S3 Table

No	Abbreviation	Phenotype
1	GDTS	Growing degree days to silking
2	GDTA	Growing degree days to anthesis
3	GASI	Growing degree days to anthesis-silking interval
4	DTS	Days to silking
5	DTA	Days to anthesis
6	ASI	Anthesis silking interval days
7	PH	Plant height
8	EH	Ear height
9	PE	Plant height minus ear height
10	EP	Ear height divided by plant height
11	PHD	Plant height divided by days to anthesis fag leaf width

Table S3. A list of 11 quantitative traits in the maize data used in this study.

S4 Table

No	Abbreviation	Phenotype	No	Abbreviation	Phenotype
1	OBM	Obesity.BMI	11	BChl	Biochem.Chloride
2	OBo	Obesity.BodyLength	12	BGl	Biochem.Glucose
3	OSS	Obesity.Date.StudyStartSeconds	13	BHD	Biochem.HDL
4	ODS	Obesity.Date.StudyDay	14	BLD	Biochem.LDL
5	OEn	Obesity.EndNormalBW	15	BSo	Biochem.Sodium
6	BAl	Biochem.Albumin	16	BTC	Biochem.Tot.Cholesterol
7	BAL	Biochem.ALP	17	BTP	Biochem.Tot.Protein
8	BLT	Biochem.ALT	18	BUr	Biochem.Urea
9	BAS	Biochem.AST	19	BEn	Biochem.EndNormalBW
10	BCa	Biochem.Calcium	20	BAg	Biochem.Age

Table S4. A list of 30 quantitative traits in the mouse data used in this study.

S5 Table

No	Abbreviation	Phenotype	No	Abbreviation	Phenotype
1	TSLL	Top second leaf length	7	FNL	Fringe neck length
2	TSLW	Top second leaf width	8	MSPW	Main stem panicle weight
3	MSH	Main stem height	9	PGW	Per plant grain weight
4	MSW	Main stem width	10	HKW	Hundred kernel weight
5	MSPL	Panicle length of main stem	11	MSSN	Spikelet number of main stem
6	MSPD	Panicle diameter of main stem	12	SGN	Grain number per spike

Table S5. A list of 12 quantitative traits in the millet data used in this study.

2 SUPPLEMENTARY DATA

S1 Data

The result of the mean squared error for all phenotypes from five species is given in the file "Table 1.XLSX".

S2 Data

The result of the overfitting for all phenotypes from five species is given in the file "Table 2.XLSX".

S3 Data

The result of the non-inferiority test for all phenotypes from five species is given in the file "Table 3.XLSX".

S4 Data

The result of the correlation coefficients for all phenotypes from five species is given in the file "Table 4.XLSX".

S5 Data

The performance results using RKHS from five species are given in the file "Table 5.XLSX".

S6 Data

The performance results using the bagging regressor from five species are given in the file "Table 6.XLSX".
